# Numerical Simulation of a Bird-Inspired UAV Which Turns Without a Tail Through Proverse Yaw †

**DOI:** 10.3390/biomimetics10040253

**Published:** 2025-04-21

**Authors:** Wee-Beng Tay, Timothy Shawn Jie-Sheng Chong, Jia-Qiang Chan, Woei-Leong Chan, Boo-Cheong Khoo

**Affiliations:** 1 Temasek Laboratories, National University of Singapore, T-Lab Building, 5A, Engineering Drive 1, #02-02, Singapore 117411, Singapore; 2 Department of Mechanical Engineering, National University of Singapore, 10 Kent Ridge Crescent, Singapore 119260, Singapore; timchongjs@gmail.com (T.S.J.-S.C.); mpekbc@nus.edu.sg (B.-C.K.); 3 Department/Institute of Aeronautics and Astronautics, National Cheng Kung University, No. 1, University Road, Tainan City 701, Taiwan; woeileong_chan@gs.ncku.edu.tw

**Keywords:** 6DOF, OpenFOAM, bell-shaped spanload, proverse yaw, CFD, UAV, tail-less, bio-inspired

## Abstract

This study numerically explores a bird-inspired tail-less unmanned aerial vehicle (UAV) design which can turn through proverse yaw by using a bell-shaped spanload wing configuration. The research methodology consists of two phases. In the first phase, the objective is to use computational fluid dynamics (CFD) simulations to validate that the bell-shaped spanload wing configuration produces proverse yaw, instead of adverse yaw, similar to other typical wing configurations. This allows the UAV to turn without a tail. The solver used is OpenFOAM and a special self-written routine is used to allow the grid to move together with the UAV, which has six degrees-of-freedom (6DOFs) to translate and rotate when its ailerons deflect after reaching steady motion. In the second phase, we investigate the effect of the sweep angle on the proverse yaw. Results show that proverse yaw is indeed produced due to the bell-shaped spanload wing configuration, as CFD simulation shows the UAV turning after aileron deflection. The effect of the sweep angle is more profound on the proverse yaw as simulations show that increasing the sweep angle by 10° increases the turning effect slightly, but decreasing it by 10° instead results in adverse yaw. These findings will have important implications for improving aircraft efficiencies and the development of wing designs.

## 1. Introduction

There has been growing adoption of unmanned aerial vehicles (UAVs) across diverse sectors. Most of these aircrafts use elliptical spanload distributions [1] which requires a rudder for coordinated turning, adding complexity and weight. As many studies have shown, we can look to nature for inspiration. These inspirations include designing flapping wing robots based on pigeons [2] and a non-flapping UAV with wing and tail morphing capabilities which is modelled after the Great Black-Backed Gull [3]. Similarly, our observations of bird flight suggest an alternative approach to turning should be possible without a rudder, as many bird species achieve efficient turning without such auxiliary yaw control surfaces.

**Figure 1:**
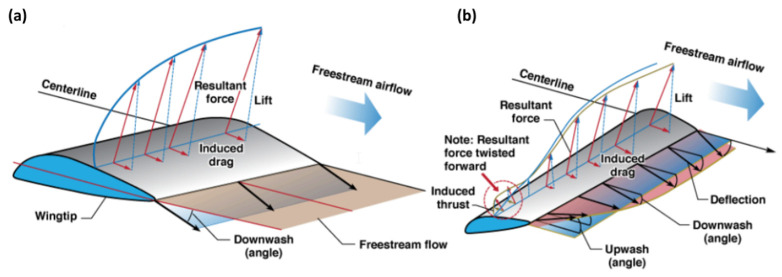
(**a**) Elliptical and (**b**) bell-shaped spanload distributions, from [5].

In a more recent paper by Prandtl [4], he proposed the bell-shaped spanload distribution, which offers a promising alternative solution, demonstrating superior efficiency compared to conventional designs for equivalent structural requirements [5], as illustrated in Figure 1. A key advantage of this distribution is its inherent generation of proverse yaw, enabling turns without additional control surfaces and thereby reducing weight while improving aerodynamic efficiency. Compared to traditional elliptical wing loading and with a 22% longer wingspan, there is an 11% increase in efficiency while maintaining minimal drag. It has to be noted that the weight of the wing may increase due to increasing wingspan, but the weight of the overall aircraft may decrease when the tail is removed due to the use of proverse yaw.

In 2016, Bower et al. [5] performed flight tests using remote-controlled aircrafts to demonstrate the concept of proverse yaw. Similarly, Newton [6] applied parameter estimation methods on the flight data from the Prandtl-2 aircraft to derive the aircraft coefficients. This allowed him to reconcile between the mathematical models and experimental data to prove the existence of proverse yaw. In connection to what is mentioned earlier about birds turning without a rudder, this shows that there are perhaps some similarities between bird’s wings and the wing with a bell-shaped spanload distribution. However, actual flight tests can be complicated to analyze with many external factors such as wind gusts affecting the final result. On the other hand, the topic of different lift distribution has been investigated by Hunsaker et al. [7] based on the lifting-line theory. Results show that for all distributions in this class except the elliptic one, an antisymmetric twist distribution can be added to control adverse yawing during roll maneuvers. Moreover, by applying specific antisymmetric twist distributions to a wing with a bell-shaped lift distribution, designers can further enhance this natural tendency and precisely control the roll–yaw coupling. With respect to numerical simulations, there have also been several studies [8,9,10,11] using software like OpenVSP v1.3 and StarCCM+ 19.04.007(2406). Yoo [8] used OVERFLOW, a Reynolds-averaged Navier–Stokes (RANS) CFD solver to determine the stall angle and flow separation angle of attack, but there is no detailed CFD analysis on proverse yaw. Kelly [11] applied Prandtl’s bell-shaped spanload to a sailplane wing. He used both a low-fidelity Vortex Lattice Method (VLM) and high-fidelity STAR-CCM+ for the investigation; results from both simulations are consistent with one another. However, Kelly’s research was mainly focused on the drag reduction aspect; hence, the simulations results did not verify the presence of proverse yaw. Nonetheless, the lower drag as predicted by Prandtl was confirmed for a bell-shaped spanload. Richter et al. [9] designed a straight tapered flying wing Biomimetic Aircraft and investigated the effect of various parameters such as the taper ratio and wing chord on proverse yaw. A low-fidelity solver, OpenVSP, based on the VLM, is used. Their final design with 10% more span and 10% more twist at the wingtips, has 16 times more proverse yaw control power than the original. Lastly, Weekley [10] focused on the design and analysis of the bell-shaped spanload using a low- (AVL, XFLR5) and high- (Star-CCM+) fidelity solver. For the banked turn simulation, unsteady CFD RANS is first used, and then steady RANS is used subsequently. He concluded that the creation of proverse yaw is evident for the wing designs in which the ailerons are engulfed in the upwash. The positive yaw-to-roll ratio and positive yaw moment also indicated the presence of proverse yaw.

The different studies described earlier use theories, experiments, and numerical simulations. In this paper, our objective is to investigate the proverse yaw characteristics using a full six degrees of freedom (6DOFs) with dynamic mesh motion, to better understand the underlying aerodynamic principles. We aim to add weight to this topic and confirm the growing literature on wings that produce the bell-shaped spanwise lift distribution. The ailerons will deflect to create a roll moment, and it can be validated through 6DOFs if proverse yaw will occur. This scenario is much more realistic, and it is not affected by external influences such as gusts. Moreover, we would also like to investigate the effect of the sweep angle on the proverse yaw. This will allow one to design a more efficient and controllable UAV.

## 2. Numerical Methods

### 2.1. Solver

OpenFOAM (OF) [12], the numerical solver used in this study, is a widely-adopted open-source CFD package that uses the finite volume method to solve the Navier–Stokes equations. It offers extensive capabilities including various numerical schemes, turbulence models, and parallel computing support through MPI for both compressible and incompressible flows. For this research, we employed pimpleFOAM, a transient solver designed for incompressible, turbulent Newtonian flows. This solver implements the PIMPLE algorithm—a hybrid of PISO [13] and SIMPLE [14] methods—which enables larger timesteps while maintaining enhanced stability and computational efficiency compared to PISO alone. PimpleFOAM also supports dynamic mesh adaptation and topology modifications. The version used is v2206.

### 2.2. Grid Motion for 6DOFs Simulations

In the current paper, the UAV must do 2 things. Firstly, it must rotate both its ailerons to create roll moment. This is usually accomplished through grid deformation in the region around the ailerons. Secondly, it must move freely under the influence of 6DOFs. Overset grids [15] offer a viable solution, using a foreground grid for the UAV and a stationary background grid. This approach maintains grid quality during the motion and can accommodate multiple moving bodies. However, as the UAV has to cover considerable distances, the background grid will have to be large enough to encompass its motion. This will make the simulations computationally expensive.

A solution to address this issue is to implement a moving grid system that tracks the aircraft’s motion and moves together with it. Kumar [16] wrote a new sixDoFDynamicMotion class code for OF v1812 which is a subset of the solidBodyMotionFunction class. This custom-developed routine allowed grid movement in conjunction with 6DOFs simulations in an overset grid. It is modified in this work to allow it to work with a moving grid and OF v2206, which is the OF version used in this study. Hence, instead of a fixed domain in most CFD simulations, the current domain follows the motion of the body of interest. In the current study, the UAV is moving and making a banked turn. Using a moving domain can result in substantial saving in computational resources as there is no need to create a large, fixed domain. The new code is known as moving_sixDoFDynamicMotion (https://github.com/zonexo/moving_sixDoFDynamicMotion, accessed on 31 January 2025).

### 2.3. Grid Convergence Study

**Figure 2:**
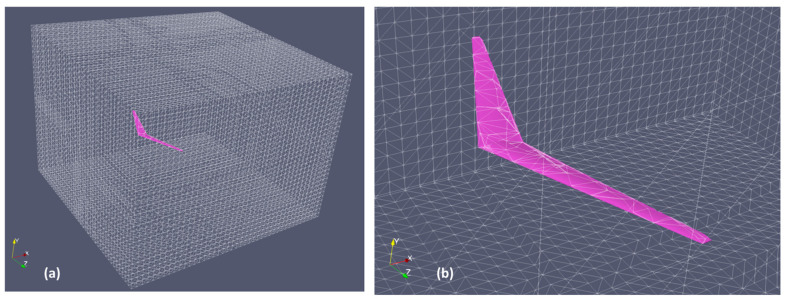
(**a**) Grid with the (**b**) enlarged UAV.

For the grid convergence study, 4 grid sizes (3.9, 6.4, 12.1, and 22.5 million) are selected. The simulation is an unsteady run at an angle of attack (AoA) of 7° using pimpleFoam, a transient incompressible solver in OF. It can accommodate both moving meshes and changes in mesh structure. The grid and the enlarged UAV can be seen in Figure 2.

Results from Table 1 show that both grids of 12.1 and 22.5 million cells give similar results. Hence a grid size of 12.1 million cells will be used for subsequent simulations. However, the grid used for subsequent simulations will be one with 11 million cells because part of the wings is removed to allow for grid deformation of the ailerons. More details will be given in the methodology section.

### 2.4. Validations

In this study, we validate our results with the experimental results from Bowers et al. [17]. It must first be clarified that the definition of AoA in Bowers et al. seems to the starting AoA after the twisting, as given in their earlier paper [5]. Hence, their AoA = 0° corresponds to our 7°. Next, the model used in the wind tunnel has a wingspan of 1.83 m, which is half of our current design. From the comparison of the CL and CD in Table 2, we can observe that the CL results match very well. However, our predicted CD is lower than the experimental result. This can be expected as the Re of our current design is higher, resulting in lower form drag. The CD is more sensitive to Re change as compared to CL.

### 2.5. Experimental Validation of the Moving_sixDoFDynamicMotion Solver

**Figure 3:**
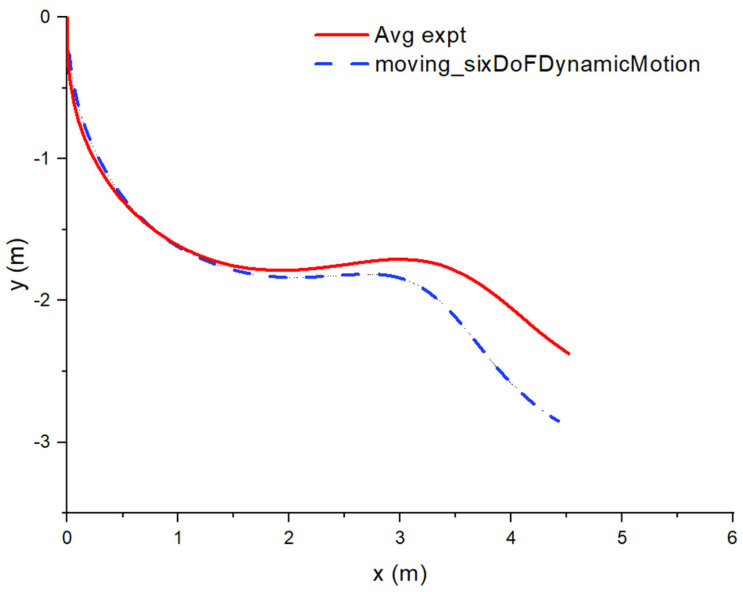
Trajectory comparison between experiment and current solver using moving_sixDoFDynamicMotion.

The new moving_sixDoFDynamicMotion solver is validated against the free fall experiment of a model aircraft found in the paper by Tay et al. [18]. In the experiment, a foam model aircraft with its nose pointing down is dropped vertically from a certain height around ground and allowed to move freely. Due to the setup of the experiment, the solver is simplified to only allow motion in the xy plane and rotation in the z direction. This is exactly the same as the validation of the MG6DOFM solver in this paper [18]. From Figure 3, it can be observed that its trajectory is similar to the experiment, with minor differences towards the latter half. As mentioned in the paper, the discrepancies in the latter part of the trajectory may be due to minor errors in the measurement of elevator angle, moment of inertia, and center of gravity. It was found that an elevator change as small as 0.5° can result in a distinct difference in the aircraft’s trajectory. More details about the experiment can be found in the paper by Tay et al. [19].

### 2.6. Simulation Setup

**Figure 4:**
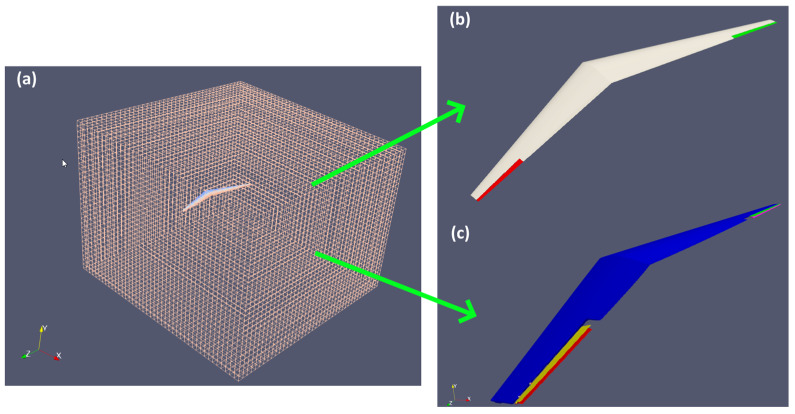
(**a**) Simulation domain with close-up view of the (**b**) original and (**c**) aileron-modified UAV.

The specifications of the UAV with a bell-shaped spanload distribution are obtained from the paper by Bowers et al. [5]. Based on the paper, the root AoA of the UAV is 7.7°. The specification is given in Table 3. The CAD is drawn in Solidworks v2018 and then exported to ANSYS workbench v2020 for surface meshing. ANSYS workbench can automatically create a good-quality surface meshed body with minimal manual interference. Regions of high curvature have a larger concentration of cells compared to low curvature regions. It is then exported to the STL format. SnappyHexMesh from OpenFOAM is then used to generate the external flow domain as we have more experience using it to generate similar domains. The grid domain with the close-up view of the original UAV on the top right is shown in Figure 4. The domain size is 8.5 × 6 × 8 m. The y+ value is 3, whereby the first grid cell has a thickness of 4.5 × 10^−5^. Due to the twist of the wing, it is very difficult to achieve y+ = 1. SnappyHexMesh will fail and no boundary layer will be generated if y+ is reduced to 1.

In the simulation, the ailerons are prescribed to rotate 7° in opposite directions to enable roll motion. The rotation of the ailerons is made possible through mesh deformation in the region around it. In OpenFOAM, the mesh motion solver used is the displacementLaplacian solver, which calculates mesh motion by solving a Laplace equation for point displacements. In the original configuration, the ailerons are directly adjacent to the wing. It is not possible to rotate the aileron while maintaining acceptable grid quality due to the close distance between the two. Hence, the proposed solution is to have a small gap of 0.12 m between the wing and the aileron on each side. In this way, the grid in the region has the flexibility to deform while maintaining grid quality. The bottom right diagram in Figure 4 shows the UAV with the modified ailerons in the neutral and magnified 3X rotated positions. Hence, the length of the ailerons are reduced by approximately 17%.

All sides of the domain use the zero gradient boundary condition. Using the specification in Table 3, the Reynolds number (Re) is 530,000. More information can be obtained from Bowers et al. [5]. For external flow, at Re > 500,000, flow is mostly turbulent, hence the kOmegaSST turbulence model was used in the simulations. The Courant–Friedrichs–Lewy (CFL) number is fixed at 1 to ensure accuracy. The simulations run on the High-Performance Computing (HPC) clusters running 360 to 1024 processors in parallel. The CPUs used include the AMD EPYCTM 7713 and the Intel 8452Y. One limitation of the current setup is that it is not able to resume from where it stops. However, different HPC clusters impose different runtime limits ranging from 3 to 7 days. Hence, all the 3 simulations are only able to reach time instants from 3.8 to 4.3 s. Nevertheless, this is sufficiently long to observe the resultant yaw. For comparison between UAVs of different sweep angles, we limit our comparison up to t = 3.8 s as one of the cases stops at this time instant.

## 3. Research Methodology

In this section, we outline the methodology used to investigate the proverse yaw characteristics using full 6DOFs with OpenFOAM and also the effect of sweep angle on proverse yaw.

### 3.1. Validation of Proverse Yaw Through Full 6DOFs Simulation

One of the important aspects of designing this simulation is to ensure that the simulation is as realistic as possible, which involves the UAV transiting smoothly from a steady constant velocity straight forward flight to making a turn bank. In order to do that, the simulation is separated into 2 stages. In the first stage, the UAV together with the grid moves in a prescribed manner at a constant speed of 19.85 m/s. This stage is supposed to mimic the UAV flying at a constant velocity, and the simulation is supposed to run for 1 s, but it is found that it has achieved a constant lift and drag after 0.4 s. In the second stage, the left and right aileron each rotates in the opposite direction at a rotation speed of 7.7°/s, causing the UAV to roll. The moving_sixDoFDynamicMotion solver is activated and the UAV’s subsequent motion is determined by its 6DOFs. There is no restriction on the translation and rotation. The maximum aileron rotation angle is 7.7°, and so the rotation will end after 1 s. Hence, from the result, it will be possible to determine if proverse or adverse yaw will prevail. There is no other external disturbance. In comparison to the standard simulation whereby the UAV is fixed with an incoming velocity, similar to a wind tunnel setup, the current simulation is a more accurate form of simulation, as it involves 6DOFs, and hence, the UAV will translate and rotate. If the UAV is fixed in a domain, it is not possible or very difficult for the incoming velocity to change its direction and magnitude due to the 6DOFs.

### 3.2. Effect of Sweep Angle on Proverse Yaw

In this section, we aim to investigate the effect of the wing sweep angle on proverse yaw. In a UAV, the design of wing sweep angle is usually for stability considerations. Any tail-less plane design would have applied a wing sweep such that the tip-end of the wing acts like a horizontal tail, providing downward force and hence balancing moment for longitudinal stability. To accomplish that, the tip-end would have to be twisted such that it is generating a downward force. On the other hand, birds do not need to sweep their wings in cruise for stability because they have horizontal tails. So, in the wing-only configuration, a bell-shaped lift distribution serves two purposes: proverse yaw and longitudinal stability.

**Figure 5:**
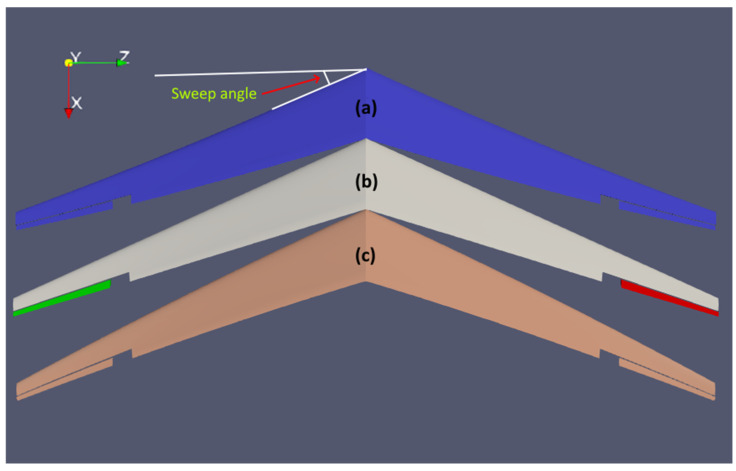
UAVs with different sweep angles: (**a**) +10°, (**b**) original, and (**c**) −10°.

In the current research, we will rotate the sweep angle by increasing and decreasing it by 10°, with respect to the original UAV. Factors such as proverse yaw, lift, and drag will be investigated. The results will be important for future UAV designs which can benefit from the bell-shaped spanload wing configuration. Figure 5 shows the UAVs with different sweep angles.

## 4. Results and Discussions

In this section, we discuss the results obtained from the simulations outlined in the methodology section to investigate the proverse yaw characteristics using the full 6DOFs with OpenFOAM and also the effect of the sweep angle on proverse yaw.

### 4.1. Full 6DOFs Simulation of UAV for Proverse Yaw Validation

**Figure 6:**
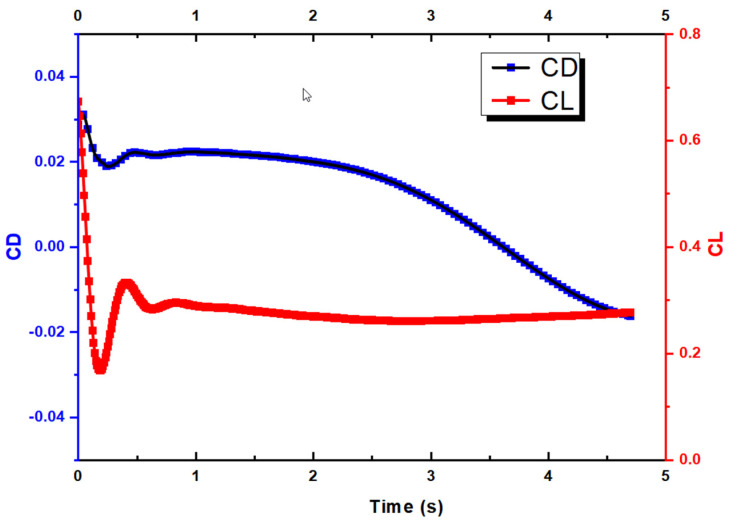
CD and CL against time for the UAV.

**Figure 7:**
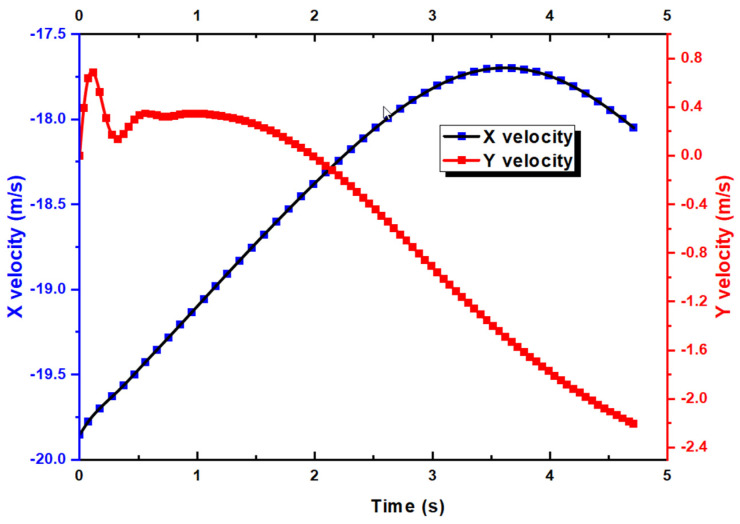
X and Y velocity against time for UAV.

**Figure 8:**
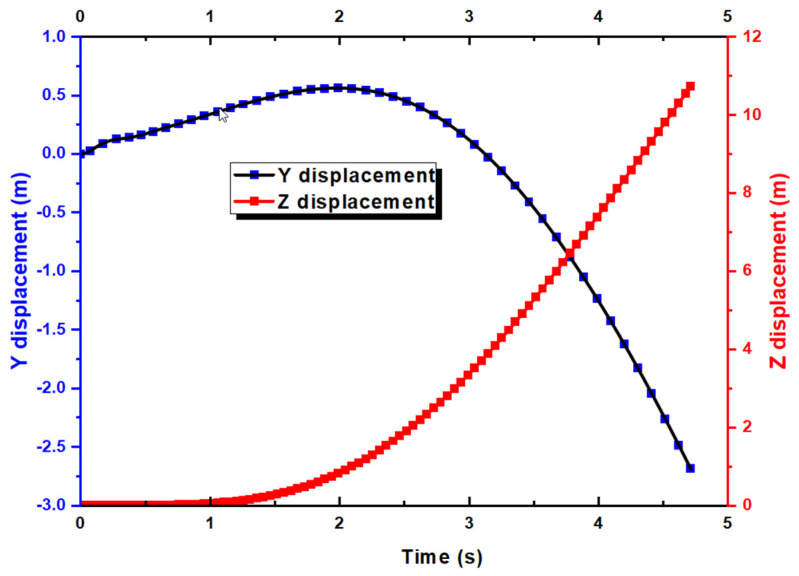
Y and Z displacement against time for the UAV.

Due to time constraints, as mentioned earlier, the simulation reached t = 4.7 s after running for 7 days on 360 CPUs in parallel. Nevertheless, this is sufficiently long for us to perform our analysis. We first look at the lift and drag coefficients over the entire duration. First of all, it must be noted that the CD and CL are calculated based on the global coordinate. As shown in Figure 6, for the f CL, it drops from around 0.65 to as low as 0.15 before maintaining at close to 0.3. Similarly, the CD decreases from 0.03 to −0.015 at t = 4.7 s. Note that due to the motion of the UAV with respect to the X axis, the X velocity is shown as negative. Figure 7 shows that the X velocity starts to decrease with a magnitude around 19.85 m/s to 17.75 m/s at t = 3.5 s, before increasing again. For the Y velocity, it has an initial increase due to the aileron deflection, which creates the roll motion and a minor speed increase for a short duration. As the aircraft rolls, it retains its forward momentum. The rolling motion temporarily reorients the lift vector, causing a component of the lift to act in the vertical direction, which can briefly increase the vertical velocity. Thereafter, the Y velocity decreases. Figure 8 shows the Y displacement of the UAV, which shows the increase in height in the Y direction up to t = 2 s, due to the increased Y velocity. After t = 2 s, the Y position of the UAV also decreases as the forward momentum diminishes.

It may seem counter-intuitive initially to observe the CD decreasing until being negative, indicating the occurrence of thrust. However, there can be a number of reasons. In a coordinated turn, the UAV’s lift vector tilts and provides a component of force in the forward direction. If the angle of attack and sideslip conditions change significantly, the aerodynamic forces can produce a net force component in the forward *x*-direction, effectively acting as aerodynamic thrust. Transient flow effects, such as circulatory forces and added mass effects, could momentarily generate forward-directed forces that act like thrust. Lastly, in a banked turn, if the UAV yaws due to asymmetric lift or differential drag on the wings, the side force in the spanwise (*z*-direction) can couple with the UAV’s velocity vector and create a forward force component. This means that the side force projected onto the flight path can contribute to a net forward acceleration.

**Figure 9:**
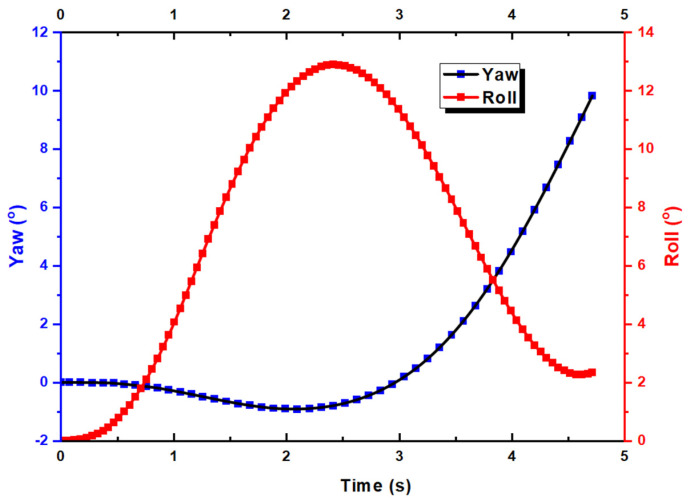
Yaw and roll against time for the UAV.

From Figure 8, one can also observe the movement of the UAV in the Z direction, indicating the effect of proverse yaw. To confirm this observation, we turn to the yaw and roll results, as shown in Figure 9. At the start of the simulation, there is a slight decrease in the yaw angle while the roll angle is decreasing. This is known as adverse yaw, which usually happens in a typical aircraft. Hence, there is a need for a vertical stabilizer. However, this only happens for around 2 s with a small yaw angle of −1° before it increases together with the roll angle, hence producing proverse yaw. In other words, from the simulation, we validated the existence of proverse yaw. From Figure 9, we also observe that the roll angle starts to decrease after reaching a maximum of 13°, which is due to roll damping and the dihedral effect. The rolling motion itself creates aerodynamic damping forces that oppose the roll, which increase with the roll rate, eventually balancing the rolling moment from the ailerons. Moreover, the UAV has dihedral wings, and the rolling motion induces sideslip, which generates a restoring rolling moment due to the difference in angle of attack between the two wings. This shows that the UAV has good roll stability as it will return to neutral position without external influence.

**Figure 10:**
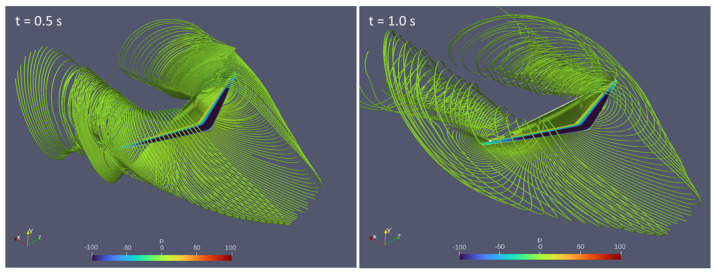
Streamlines with pressure contour at t = 0.5 and 1.0 s.

We also visualize the streamlines with pressure contours at t = 0.5 and 1.0 s, as shown in Figure 10. It can be observed that the two vortex tubes are located about 70% from the wing root on both sides. In comparison, typical vortex tubes for swept back wings tend to be nearer the wingtips. However, in this case, the airflow over the upper surface of the wing separates, creating regions of low pressure and swirling flow that roll up into vortices. The primary vortex (the stronger and more dominant vortex) forms near the 70% span location as the pressure gradient is strongest in this region, driving the flow separation and vortex formation. The spanwise flow carries the separated flow outward from the wing root, but the vortex tends to stabilize and concentrate around the 70% span location rather than continuing all the way to the wingtip. Comparing against Figure 1, we can observe that the bell-shaped spanload distribution is rather different from the elliptical distribution. This could have resulted in the local induced drag being higher in this 70% region compared to further inboard or outboard. In a banked turn, the outer wing moves faster than the inner wing due to the turn radius difference. Since induced drag is roughly proportional to lift squared, the outer wing (which is moving faster) produces more forward-directed induced effects than the inner wing. This creates a net yawing moment in the direction of the turn, resulting in proverse yaw. Moreover, it is also mentioned in the paper by Hammer and Garmann [20] that for UAVs with a bell-shaped spanload wing configuration, the tip vortex is weak at low AoAs. This has also been mentioned by Bowers et al. [5] and explains why birds flying in formations position themselves directly behind the vortex roll-up.

As mentioned earlier in the simulation setup section, the length of the ailerons have been reduced by approximately 17%. The purpose of ailerons is to produce an unbalanced side force to create a banked turn. Since both sides of the ailerons’ length have been reduced by the same amount, its effect would be similar to one while using full-length ailerons. However, the unbalanced force created by the full-length ailerons will be greater, and it will create a stronger roll moment and possibly stronger proverse yaw. Hence, the aileron modification will not change the current result.

### 4.2. Effect of Sweep Angle on Proverse Yaw and UAV’s Performance

Having successfully validated the existence of proverse yaw, we move on to investigate the effect of the sweep angle. As mentioned earlier in the methodology, the sweep angle has been increased and decreased by 10%. These two new cases are termed the s10 and s-10 UAV, respectively. The original UAV is now termed the s0 UAV for clarity. Due to the variation in computational loads, the s10 and s-10 UAV cases reach a simulated time of 4.6 and 3.8 s. Nevertheless, these timings are sufficient for our analysis.

**Figure 11:**
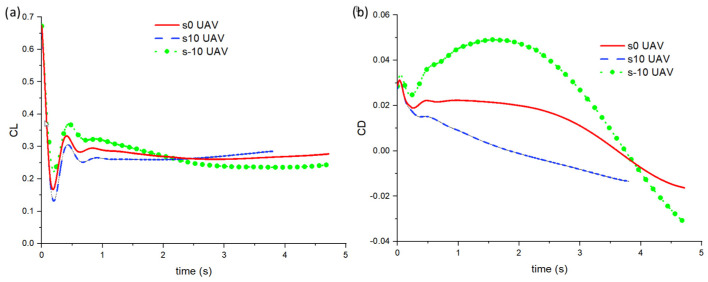
(**a**) CL and (**b**) CD against time for the s-10, s0, and s10 UAVs.

**Figure 12:**
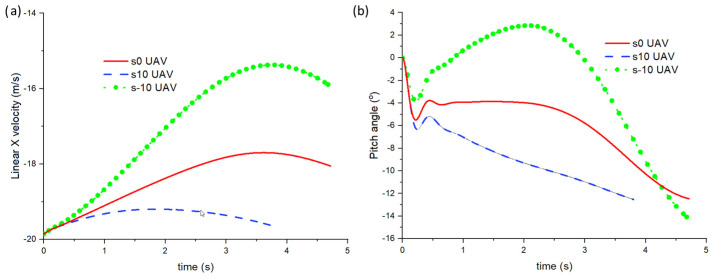
(**a**) Linear X velocity and (**b**) pitch angle against time for the s-10, s0, and s10 UAVs.

Figure 11 shows the CL and CD against time plots for the UAVs. Due to the sweep angle, the pitch angle, as shown in Figure 12, undergoes changes, and this directly affects the CL and CD. One can observe that the shape or the pitch plot is very similar to the CD plot in Figure 11b. However, as the simulation proceeds, it can be observed that the CL for the s10 UAV is higher than the other 2 cases. In terms of the CD, the s-10 UAV case sees an increase to 0.05 before decreasing pass zero, effectively gaining thrust. As explained earlier in the previous section, this is possible although it may seem counter-intuitive initially. For the other two cases, both also decrease and later gain small thrust towards the end of their simulations. Hence, as mentioned earlier, as the UAVs turn, their pitch angle changes. A higher pitch angle (nose pointing upwards) indicates higher drag and, therefore, a larger decrease in the forward linear X velocity, as seen in the s-10 UAV case. When the pitch angle is around 10°, thrust is generated, and it can be observed in the slight increase in forward speed in all cases.

**Figure 13:**
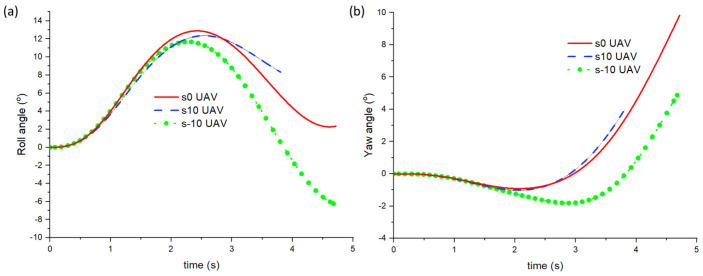
(**a**) Roll and (**b**) yaw angle against time for the s-10, s0, and s10 UAVs.

**Figure 14:**
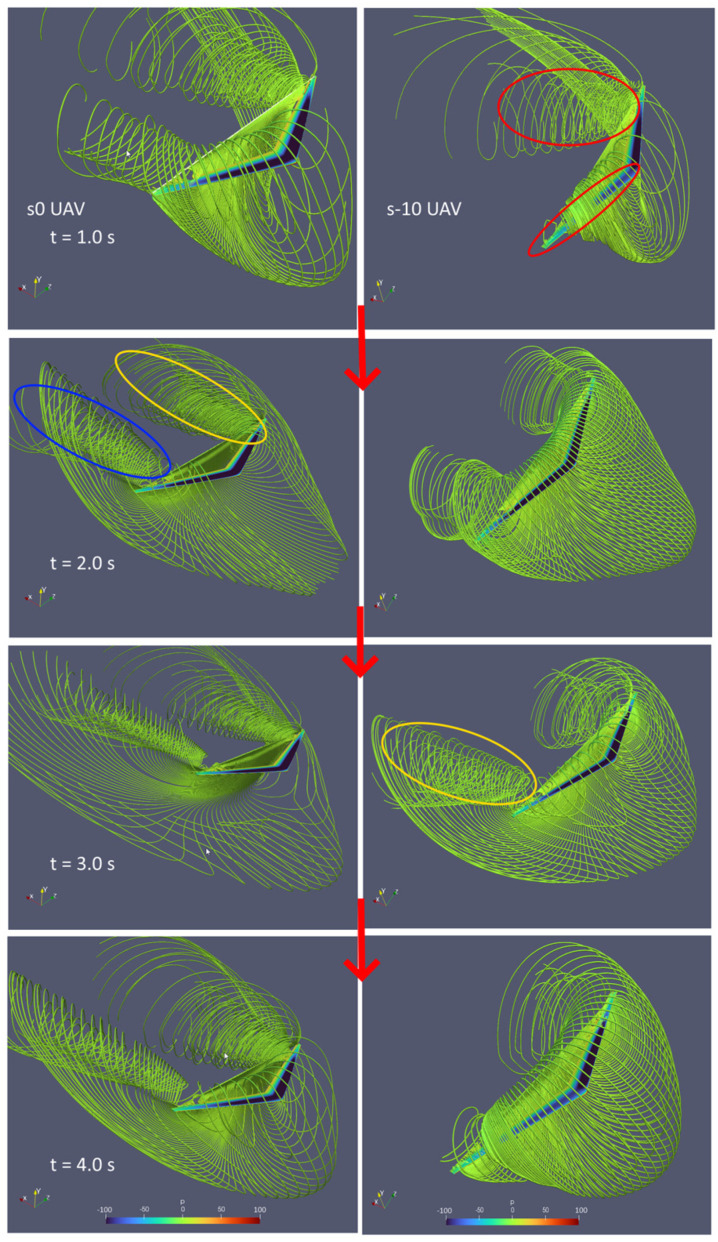
Streamlines with pressure contour from t = 1.0 to 4.0 s for s0 (**left**) and s-10 (**right**) UAV cases.

Next, we compare the roll and yaw angle for the different UAV cases. As shown in Figure 13, the roll angles for all cases are very similar up to around t = 2 s. They all increase to a maximum of between 11 and 13° and then start to decrease. One can observe that for the s-10 UAV case, the roll angle changes from positive to negative after 3.8 s. Similarly, all cases have the same yaw angle up to t = 2 s, after which both the s0 and s10 UAV cases follow similar yaw angle changes. However, the s-10 UAV case instead exhibits adverse yaw with a positive roll + negative yaw angle up to t = 3.8 s and then switches to a negative roll + positive yaw angle. In other words, the s-10 UAV case does not obtain proverse yaw. On the other hand, increasing the sweep angle exhibits minor difference to the yaw angle, and it seems that the yaw angle experienced by the s10 UAV is slightly more than that of s0. This shows that increasing the wing’s sweep angle increases the proverse yaw effect, albeit only by a small amount. Due to the runtime limitation, we are only able to show the yaw angle of the S10 UAV case up to 3.8 s. At t = 3.8 s, the yaw angles of the s-10, s0, and s10 UAVs are −0.1°, 3.3°, and 4.0°. However, we can observe that the trend of the s10 UAV’s roll and yaw plots follow that of the s0 UAV’s plots closely. This indicates that the yaw angle of the s10 UAV will most likely reach around 11 to 12° at t = 4.6 s. On the other hand, the roll angle of the s10 UAV does not decrease as fast as the s0 UAV case. If we compare the streamlines between the s0 (left) and s-10 (right) UAV cases as shown in Figure 14, we can observe two very distinct vortical patterns. At t = 1.0 s, strong vortex tubes can be seen at around a 70% wingspan from the root for the s0 UAV case. However, there is only a single vortex tube on the left wingspan side for the s-10 UAV. Moreover, the vortex tube is directed along the wingspan. Both these features have been circled in red. At t = 2.0 s, both cases have similar vortex tubes on both sides of the wingspan, but the s0 UAV case has much more concentrated vortex tubes, as shown by the blue circles. At t = 3.0 s, there is minimal visual change in the s0 UAV case, but for the s-10 UAV case, the right side of vortex tube seems to have increased in strength compared to the left side, as shown by the orange circle. At t = 4.0 s, the left vortex tube of the s0 UAV case has become less concentrated and hence a reduction in strength is observed. This causes an imbalance between the forces on the left and right, resulting in a yaw towards the left, as observed in Figure 13b. For the s-10 UAV case, the vortex tube on the left has weakened in strength while the one on the right is now directed towards the wingspan.

## 5. Conclusions

In this study, we modify an existing OpenFOAM custom subroutine to allow for 6DOFs with moving grid simulations. This unique capability allows us to validate whether proverse yaw is possible in a UAV with a bell-shaped spanload distribution. The results show that proverse yaw can be achieved with this wing configuration. Moreover, the effect of sweep angle on the performance of the UAV is also investigated. It is found that increasing the sweep angle by 10° produces a slightly greater proverse yaw effect compared to the original s0 UAV case. However, decreasing the sweep angle by 10° instead produces adverse yaw, similar to aircraft with elliptical spanload distributions. This study proves conclusively the existence of proverse raw. Hence, designs of future UAVs or aircraft with a bell-shaped spanload distribution can eliminate the tail, which can reduce the overall weight and simplify their overall design. It also shows the importance of the wing sweep angle in the design of future UAVs or aircraft who would like to make use of proverse yaw to turn without a tail. As only ±10° relative to the original sweep angle is investigated, there is potential that further increase in the sweep angle can increase the proverse yaw effect further. Moreover, the efficiency of the bell-shaped spanload distribution is supposed to be even better than the elliptical one, and it is worth investigating further. In the current study, the ailerons are reduced by approximately 17% to allow for grid deformation. A new grid deformation algorithm may be able to maintain grid quality without length reduction, and this can provide more accurate results on the magnitude of proverse yaw. Lastly, the custom subroutine for OpenFOAM can also be used in a variety of problems involving 6DOFs and a moving body. The source code for the moving_sixDoFDynamicMotion can be downloaded on Github. The address is https://github.com/zonexo/moving_sixDoFDynamicMotion (accessed on 31 January 2025).

## Figures and Tables

**Table 1:** CD and CL for different grid sizes.

Grid Size (Millions)	CD	CL
3.9	0.61	0.54
6.4	0.031	0.66
12.1	0.031	0.69
22.5	0.029	0.71

**Table 2:** Comparison of CD with different solvers.

Variable	Bowers et al. [17] Wind Tunnel at AoA = 0°	Current at AoA = 7°
CL	0.71	0.71
CD	0.043	0.29

**Table 3:** Specification of the UAV.

Center of mass	(0, 0, 0) m
Mass	6.58 kg
Roll inertia	7.355 kgm^2^
Yaw inertia	7.888 kgm^2^
Pitch inertia	0.368 kgm^2^
Wingspan	3.749 m
Centerline chord length (reference length)	0.4 m
velocity	(19.85, 0, 0) m/s
Planform area (reference area)	0.94 m^2^
Dihedral angle	2.5°
Leading edge sweep	24° at the nose
Aileron location	Located in the outboard 14% of each wing, in the trailing 25% of the chord

## Data Availability

The author declares that the source code for the moving_sixDoFDynamicMotion will be shared on Github once this manuscript is published.

## Abbreviations

The following abbreviations are used in this manuscript:

6DOF	6 degree-of-freedom
CD	Drag coefficient
CL	Lift coefficient
OF	OpenFOAM
